# The Effects of Intestinal Nematode L4 Stage on Mouse Experimental Autoimmune Encephalomyelitis

**DOI:** 10.1007/s00005-017-0489-z

**Published:** 2017-10-03

**Authors:** Katarzyna Donskow-Łysoniewska, Katarzyna Krawczak, Katarzyna Bocian, Maria Doligalska

**Affiliations:** 1 Department of Parasitology, Institute of Zoology, Faculty of Biology, University of Warsaw, Miecznikowa 1, 02-096 Warsaw, Poland; 2 Department of Immunology, Institute of Zoology, Faculty of Biology, University of Warsaw, Warsaw, Poland; 3 Independent Laboratory of Parasitology, General Karol Kaczkowski Military Institute of Hygiene and Epidemiology, Warsaw, Poland

**Keywords:** Helminth therapy, Nematodes, EAE, Immunomodulation

## Abstract

Helminths use various immunomodulatory and anti-inflammatory strategies to evade immune attack by the host. During pathological conditions, these strategies alter the course of disease by reducing immune-mediated pathology. The study examines the therapeutic effect of the nematode L4 stage based on an in vivo model of multiple sclerosis, monophasic encephalomyelitis (EAE), induced by sensitization with MOG_35–55_ peptide in C57BL/6 female mice infected with the intestinal nematode *Heligmosomoides polygyrus*. The EAE remission was correlated with altered leukocyte number identified in the central nervous system (CNS), and temporary permeability of the blood–brain barrier at the histotrophic phase of infection. At 6 days post-infection, when the L4 stage had almost completely attenuated the clinical severity and pathological signs of EAE, CD25^+^ cell numbers expanded significantly, with parallel growth of CD8^+^ and CD4^+^, both CD25^+^Foxp3^+^ and CD25^+^Foxp3^−^ subsets and alternatively activated macrophages. The phenotypic changes in distinct subsets of cerebrospinal fluid cells were correlated with an inhibited proliferative response of encephalitogenic T cells and elevated levels of nerve growth factor and TGF-β. These results enhance our understanding of mechanisms involved in the inhibition of immune responses in the CNS during nematode infection.

## Introduction

Helminths and, to a greater degree, gastrointestinal nematodes represent a potential new paradigm in the treatment of autoimmune human disease, including multiple sclerosis (MS). Helminths use various immunomodulatory and anti-inflammatory mechanisms to evade destruction by the host immune system and are capable of altering the course of disease (Dhingra and Dwivedi 2014). For infectious agents such as helminths, co-evolution over millions of years has developed a mutualism in which both the host and the parasite derive some benefit from their relationship. While the immunosuppression and immuneregulation induced by gastrointestinal nematodes is obviously beneficial for the parasite, it also benefits the host locally through reduced pathology in the intestinal tissue, and peripherally by the suppression of the immunity provoked by the infection; it also affects responses to other non-nematode antigens (Barthlott et al. 2003). However, although our understanding of the immunoregulatory potential of helminths and helminth-mediated attenuation of autoimmune disease remains scant, future focused therapeutic approaches to MS employing helminth-derived immunoregulators (Ruyssers et al. 2008) and the identification of therapy targets according to helminth-induced immunoregulatory pathways may prove effective. There is undoubtedly a need to develop novel and safe medications to treat MS. The treatment of autoimmune diseases improved greatly during the second half of the twentieth century; however, no known treatment for MS is fully effective, and some of the very effective immunomodulators in current use have considerable side effects such as infusion reactions, infections, malignancies and neurological disorders, which limit the benefit for a number of patients (Willrich et al. 2015).

Experimental autoimmune encephalomyelitis (EAE) is one of the most intensively studied animal models of MS, with similarities in disease susceptibility (Gold et al. 2006; Mix et al. 2008). Two hallmarks of the pathogenesis of MS and EAE are the migration and accumulation of autoreactive myelin-specific CD4 T lymphocytes in the brain and spinal cord tissue. These increases result in elevated levels of cytokines and free radical species, leading to demyelination, and eventually to impaired neuronal transmission (Geurts and Barkhof 2008). *Heligmosomoides polygyrus*, an intestinal trichostrongylid nematode naturally occurring in mice, is an excellent choice for studies of parasite immunomodulation. The parasite causes a chronic, asymptomatic infection restricted to the small intestine. The infected mice develop immunological characteristics which are very similar to those observed in hookworm (*Necator americanus*) infection in man. It has previously been demonstrated that infection with *H. polygyrus* at stage L4 promotes the recovery of mice undergoing EAE, although the mechanism is unknown (Donskow-Łysoniewska et al. 2012). The present study elucidates the effect of therapeutic nematode intervention on autoimmunity in the central nervous system (CNS).

## Materials and Methods

### Ethics Statement

All experimental procedures were performed according to the Polish Law on Animal Experimentation and EU Directive 2010/63/UE, and approved by the First Warsaw Local Ethics Committee for Animal Experimentation (ID 151/2011).

### Mice, EAE Immunization and Nematode Infection

The experiment was conducted on 8-week-old pathogen-free female C57BL/6 mice which weighed 20–25 g at the start of the study. Mice were kept at the animal house facilities at the Faculty of Biology and placed in groups of six in cages in a controlled room at a temperature of 24–25 °C and a 12/12-h lighting regimen, and with ad libitum access to drink and commercial pellet food. Animals were allowed to adjust to the laboratory conditions for a minimum of 7 days before experimental manipulation.

Groups of six mice were used in each independent experiment, and each experiment was performed at least in triplicate with similar results. All experiments contained a control groups: an uninfected group and a group only infected with nematodes. For clarity, only the results of the EAE immunized mice are presented, unless otherwise stated.

EAE was induced by subcutaneous injection in the rear flanks with 200 µg of myelin oligodendrocyte glycoprotein MOG_35−55_ (purity > 95%) per animal emulsified in complete Freund’s adjuvant (CFA) containing 300 µg of *Mycobacterium tuberculosis* H37RA strain. Immediately thereafter and again 48 h later, the mice received an intraperitoneal injection of 400 ng of *Bordetella pertussis* toxin (PTX; Sigma, St. Louis, MO, USA) in 100 µl of phosphate-buffered saline (PBS; pH 7.2). These protocols will produce moderate, non-lethal EAE. The control mice were injected only with PBS. *H. polygyrus* infection was performed 21 days post-immunization, in the acute phase of EAE when the mice showed strong EAE signs (Donskow-Łysoniewska et al. 2012). Mice were infected with 300 L3 stages or with PBS, pH 7.2, using an oral gavage tube.

### EAE Clinical Assessment

All animals were weighed and observed daily by the same two researchers in a blinded fashion. Clinical signs and ascending paralysis in EAE was assessed according to IACUC Guideline Experimental Autoimmune Encephalomyelitis and Other Demyelinating Rodent Disease Models (http://www.upenn.edu/regulatoryaffairs/Documents/iacuc/guidelines/iacucguideline-eae.pdf), on a six-stage scale: 0—clinically normal; 1—decreased tail tone or weak tail only (up to tail paralysis); 2—hind limb weakness (paraparesis); 3—hind limb paralysis (paraplegia) and/or urinary incontinence; 4—complete paralysis (tetraplegy) of hind limbs and front limbs; 5—moribund. Day 0 was considered the day of immunization. Scoring began the day after immunization and continued to the end of the experiments.

### Cerebral Spine Fluid, Brain and Blood Sample Collection

Mice were terminally anesthetized with carbon dioxide 6 days post-infection (DPI). For cerebrospinal fluid (CSF) collection, the mice were anesthetized with caution and the skin and musculature over the head and the upper back were uncovered. The head of the animal was tilted downwards at a 90° angle. An insulin syringe was inserted between the occipital protuberance and the spine of the atlas gently penetrating the atlanto-occipital membrane. The CSF was slowly aspirated, yielding approximately 40 µl of clear liquid with no blood contamination. Peripheral blood samples were taken after cardiac puncture. Erythrocyte depletion by red cell lysis was performed using red blood cell (RBC) lysis buffer. The CSF samples were centrifuged at 2000×*g* at 4 °C for 15 min. The supernatants were stored at − 80 °C. The pellets were recovered for cellular analysis or cell culture depending on the experiment. The serum obtained from heparinized blood was isolated and stored at − 80 °C, and the pellets were recovered for analysis as above. The brains were quickly dissected from the cranium and photographed. Depending on the experiment performed, the brain was stored in 10% formalin for 24 h or examined for brain cerebral edema measurement.

### Differential Cell Counts

The number and vitality of lympho-mononuclear cells were analyzed in three ways: the first using a Countess Automated Cell Counter (Invitrogen, Life Technologies, CA, USA), the second using a Muse Count and Viability Assay kit (Merck Millipore, USA) followed by a Muse Cell Analyzer (Merck Millipore, USA) in accordance with the manufacturer’s instructions, and the third using a manual count under light microscopy with Trypan blue (Gibco, Paisley, UK). The apoptosis of cells was detected with the Apoptosis Assay Kit and analyzed using a Muse Cell Analyzer according to the manufacturer’s instructions (Merck Millipore, USA).

Following this, 20-µl samples of CSF and peripheral blood smears were air-dried, then stained with Diff-Quik (Sigma/Aldrich, Castle Hill, New South Wales, Australia), according to the manufacturer’s instructions. All smears were examined with an OLYMPUS BX-60 (Center Valley, PA, USA) by a single researcher. Differential cell counts were performed at 100× magnification and images of each smear were analyzed using computer analysis. The results are expressed as the number of cells per smear.

### Histological Evaluation

For histological evaluation brains were excised and stored in 10% sucrose solution for 24 h. The specimens were then placed in increasing concentrations of sucrose solution: 20% then 30% (Sigma, St. Louis, MO, USA) over 3 days to prepare the tissue for cryosectioning. Eight-micrometer-thick sections were stained with Harris’ hematoxylin (0.1%) and eosin Y (1%) (H&E) for evaluating inflammatory cell infiltration and with 0.2% solochrome/eriochrome cyanin impregnation (Sigma, St. Louis, MO, USA) and 10 ml of 10% FeCl_3_·6H_2_O (Sigma) in 3% HCl. The sections were then washed with running tap water, followed by differentiation in 1% aqueous NH_4_OH for demyelination. Histological examination was performed as above. Images were acquired with a DXM 1200 digital camera on a Nikon Eclipse TE200 microscope and analyzed using NIS Elements F2.30 software (Nikon). Brain sections were scored for infiltration; (0—no lesions; 1—mild cell infiltrates; 2—moderate cell infiltrates; 3—severe cell infiltrates), and demyelination; (0—no demyelination; 1—mild demyelination; 2—moderate demyelination; 3—severe demyelination) by observer blinded to sample identity. The brain pathology score was calculated by adding the infiltration or demyelination scores for each animal, and averaging the result within each group.

### Brain Cerebral Edema Measurement

The wet weight of the brain was determined immediately after removal. The brain tissue was then completely dried in a 55 °C incubator until the dry weight became constant (dry weight). The percentage of the water content of the brain was determined as the difference between dry and wet weights according to the formula: brain water content = (wet weight − dry weight)/wet weight × 100% (Potter et al. 2006).

### In Vivo Magnetic Resonance Imaging

Brains were scanned with a Bruker BioSpec 70/30 Avance III (Billerica, MA, USA) spectrometer at 7 T (7T), with a transmit cylindrical radiofrequency coil (8.6 cm inner diameter) and a mouse brain-dedicated receive-only array coil (2 × 2 elements) positioned over the head. The animals were positioned prone, with the head placed in the stereotactic apparatus and an anesthesia mask, and were anesthetized with 1.5–2% isoflurane in a mixture of oxygen and air delivered via a nose cone. The animal body temperature was maintained at 37 °C by a flow of conditioned air. Respiration was monitored throughout the experiment. The scan time was approximately 1 h per animal. Localizer TriPilot scans were used for accurate positioning of the animals inside the magnet. Parametric T2 maps covering whole mouse brain were acquired with Multi Slice Multi Echo 2D sequence (TR. 3000 ms, TE 2.99 ms, echo spacing = 11 ms, spatial resolution = 172 mm × 172 mm, slice thickness = 1 mm, 15 slices, no gap). Magnetic resonance images were read blinded to the time of examination after immunization and applied by an experienced neuroradiologist.

### Cytokine, Neurotrophin and Survivin Measurement

CSF and serum were assayed for cytokines and other factors using commercially available enzyme-linked immunosorbent assay reagents for IL-17A, TNF-α, MCP-1, TGF-β and IL-10 (e-Bioscences, San Diego, USA) and IFN-γ, IL-4, IL-6 and IL-12p40 (BD Biosciences, Pharmingen, San Diego, USA), Elisa Kit for survivin (USCN Life Science Inc, China); Elisa Kit for Nerve Growth Factor NGF (USCN Life Science Inc, China); Elisa Kit For Neurotrophin 3, NT3 (USCN Life Science Inc, China) according to the suppliers’ guidelines. For the TGF-β measurement, the samples were acidified and measurements were taken of the latent and active cytokine excreted into the culture medium. The plates were read at 490 nm (cytokines) or 450 nm (factors) using u-Quant spectrophotometer (Bio-Tek, Acton, MA, USA). The mean optical densities (OD) were compared with the standard curves prepared using recombinant proteins.

### Flow Cytometry Analysis of T Cells and Macrophages

CSF leukocytes were phenotyped for T cells and macrophage surface markers by Four-Color Flow Cytometry (FACSCalibur; Becton Dickinson, Mountain View, CA, USA). Samples of 1 × 10^6^ cells were incubated with 5-μl antibodies diluted in PBS (pH 7.2) with 0.5% bovine serum albumin (BSA, Sigma, Germany) for 30 min at 4 °C. For detection of T cell markers, monoclonal IgG2a isotype control antibodies (BD Biosciences, Pharmingen, San Diego, CA, USA) were used against the following antigens (e-Biosciences, CA): rat allophycocyanin (APC)–anti-CD25, fluorescein isothiocyanate (FITC)–anti-CD8, peridinin–chlorophyll–protein complex (PercP)-anti-CD4 and phycoerythrin (PE)–anti-VLA-4 (α4β1-integrin). For staining Foxp3, cells were permeabilized in cytofix/cytoperm for 2 h, washed in perm/wash buffer (eBiosciences, CA) and stained with rat anti-mouse Foxp3 (eBiosciences, CA) for 30 min at 4 °C. The results were analyzed using LYSIS II Software (BD, Le Pont de Claix, France).

For the detection of macrophage-associated markers, the monoclonal IgG2a isotype control antibody (BD Biosciences, Pharmingen, San Diego, CA, USA) was used against the following antigens (e-Biosciences): PercP-CD11b, PE-CD124 (IL-4Rα) and APC-CCR2.

The expression of detected markers was estimated as the mean fluorescence intensity (MFI). Viable cells were analyzed. Live cells were manually gated on the basis of forward (FSC) scatter and side scatter (SSC). Immunofluorescence reactivity was determined by automated multiparameter flow cytometry analyzing at least 500,000 cells per sample gate.

### T Cell Proliferation Assay

For in vitro culture, CSF and blood cells were washed and re-suspended in complete RPMI 1640 medium supplemented with 5% heat-inactivated fetal bovine serum, penicillin (100 U/ml), streptomycin (100 μg/ml), l-glutamine (2 mM) (Gibco, Inchinnan, Scotland, UK). Cells were incubated in six replicates at 2.5 × 10^5^ cells/well of a 96-well flat-bottom plates (BD, Costar Acton, MA, USA) previously coated with anti-CD3/CD28 monoclonat antibody (2 µg/ml, BD Biosciences, Pharmingen, San Diego, CA, USA) or incubated with 5 µM of MOG_35–55_ or with complete medium alone in a total of 100 µl at 37 °C in 5% CO_2_. The optimal MOG_35–55_ concentration was determined during a preliminary study. Proliferation was evaluated after 48 h using the CellTiter 96^®^ AQ_ueous_ One Solution Reagent (Promega, USA) contains a tetrazolium compound [3-(4,5-dimethylthiazol-2-yl)-5-(3-carboxymethoxyphenyl)-2-(4-sulfophenyl)-2H-tetrazolium, inner salt; MTS]. During the last 4 h of the culture, 20 μl of MTS solution was added to each well and incubated under the same conditions. The absorbance was measured by spectrophotometry at 490 nm (u-Quant, BD, Costar, Acton, MA, USA).

The ability of lymphocytes to proliferate was calculated using the following formula: proliferation % = (OD_S_/OD_M_) × 100, where OD_S_ indicated the optical density (OD) of the cells incubated with stimulant MOG and CD3/CD28 antigen respectively and (OD_M_) indicates the OD of the control sample with medium alone. For detection of lymphocyte viability after 48 h of culture, cells were washed with PBS, re-suspended in 1% BSA (Sigma-Aldrich, USA), stained with the Muse Count and Viability Assay Kit and analyzed using a Muse Cell Analyzer (Merck Millipore, USA) in accordance with the manufacturer’s instructions.

### Evans Blue Extravasation

In a separate set of experiments, 2% Evans Blue (EB; Sigma, Austria) sterilized solution was injected slowly through the tail vein at a dosage of 4 ml/kg per animal and allowed to circulate. Thirty minutes after the injection, the mice were perfused with lukewarm saline to remove intravascular EB dye. The brains were removed, weighed and homogenized with 500 µl of PBS and mixed with 500 µl 60% treated with trichloroacetic acid to precipitate any protein. The samples were centrifuged for 30 min at 1000*g* and the supernatants were measured at 610 nm using a Q-quant spectrophotometer (Bio-Tek, Acton, MA, USA) to determine the relative amount of EB in each sample. The blue colorations of the lungs were controls for the effective tissue distribution of EB. The animals injected with EB were not destined for further examination.

### In Vivo Labeling Study

FluidMAG-D–nanoparticles (Chemicell, GMBH, Berlin)–ferrofluids was administered intravenously (i.v.) via tail vein injection 24 h prior to in vivo magnetic resonance imaging (MRI). The mice were anesthetized as above, using the anesthetic 1.5% isoflurane gas in an oxygen atmosphere, and imaged using FLASH-3D. The parameter values used for mouse brain imaging with FLASH-3D were as follows: TR/TE = 40/20 ms, flip angle = 30°, bandwidth = 21 kHz, two signal averages, the spatial resolution was 0.208 × 0.208 × 0.208 mm. After imaging, the mice were used in the post-mortem experiment. The SPIO are cleared from the circulation primarily by the reticulo-endothelial system (Bourrinet et al. 2006).

### Statistical Analyses

Quantitative data were processed using analysis of variance (one- or two-way ANOVA) using MINITAB Software (Minitab Inc., PA, USA). A *p* value < 0.05 was considered significant. Data were expressed as mean ± SD.

## Results

### *H. polygyrus* L4 Stage Reduces the Severity of Ongoing Moderate EAE

**Fig. 1:**
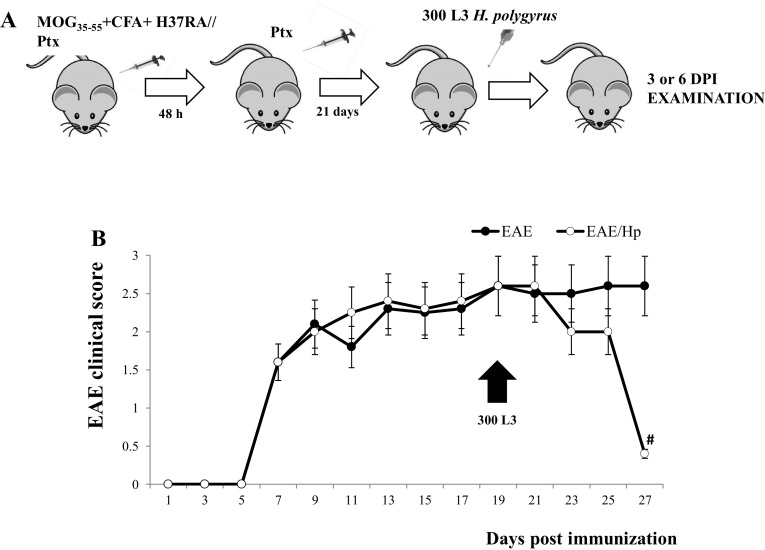
Effect of *H. polygyrus* infection on EAE in C57BL/6 females. Immunization scheme (**a**). At Day 0, the mice of the EAE group were immunized with MOG_35–55_ emulsified in CFA containing *M. tuberculosis* H37RA strain; they received *B. pertussis* toxin (Ptx) immediately afterwards and again 48 h later. On Day 21 after immunization, the mice were infected with L3 *H. polygyrus* (**b**, arrowhead). The mice were examined 3 or 6 days post-infection. Clinical characteristics of EAE after nematode treatment (**b**). The results of one representative experiment are presented as the mean ± SD of six mice per group. ^#^ *p* < 0.05 compared with EAE group assessed by ANOVA

To investigate the beneficial effect of nematode infection on the ongoing autoimmune encephalitogenic processes, all mice were monitored for disease progression until the end of the experiment at six DPI (Fig. 1a, b). In all C57BL/6 females injected with MOG_35–55_, the disease incidence was 100%. Immunized mice developed neurological signs from the seventh day after immunization in the following sequence: hind limb paralysis followed by tail paralysis. At 21 days post-immunization, the mice were infected with 300 L3 *H. polygyrus*. The control mice injected with PBS did not develop any clinical signs. The course of EAE was inhibited from the second day of infection; however, a strong recovery was observed in 100% of mice at six DPI (Fig. 1b).

### *H. polygyrus* Altered Leukocyte Number at CSF

#### In Vivo Cellular Imaging

**Fig. 2:**
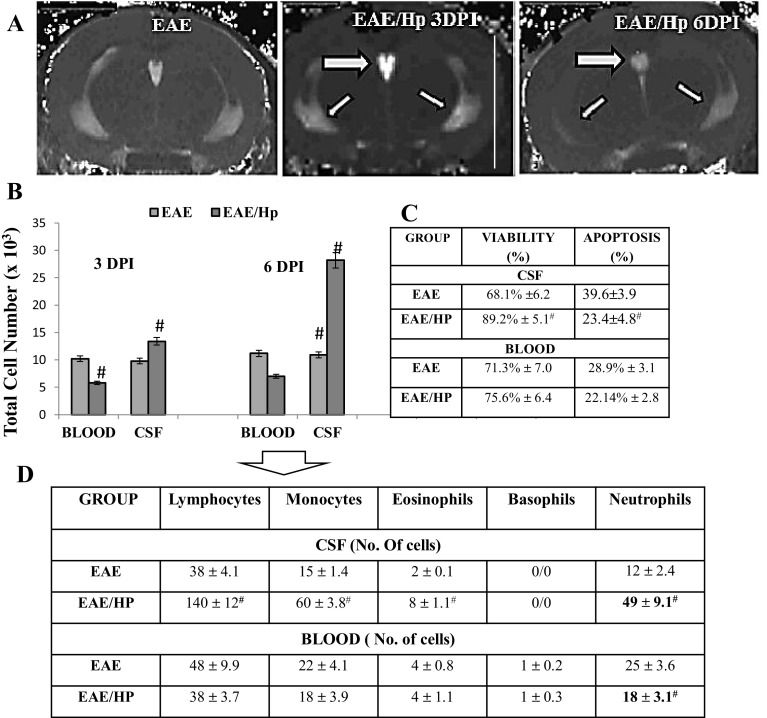
Changes in cell number in CSF and blood, 3 and 6 days post-*H. polygyrus* infection in EAE mice. MR images of EAE lesion detection 3 and 6 days post-nematode infection (**a**). A coronal T2-weighted image (TR = 3000 ms, TE = 2.99 ms, X × Y × Z = 172 mm × 172 mm × 1 mm) of the brain hippocampus depicting signal intensities in the choroid plexus (tick arrow) and lateral ventricle (thin arrows) as bright white against the darker gray neural tissue 3 and 6 days after *H. polygyrus* infection. Total cell number in the CSF and peripheral blood (per/µl) (**b**), cell viability (%) and frequency of apoptosis in the CSF and blood cells at 6 days post-infection (6DPI; **c**). Six days post-nematode infection, the leukocytes were visualized by Diff–Quik staining (**d**). The results of one experiment are represented as the mean ± SD of six mice per group. The results were analyzed using ANOVA. Statistical significance ^#^ *p* < 0.05 compared with EAE

Together with the clinical observations, MRI scans of mice were also performed. No lesions were present on T_1_-w images; however, results were seen on the corresponding T2-w images, which are considered as the gold standard in the detection of MS lesions (Bendszus et al. 2008). The coronal T2-w MRI measure for overall disease burden in EAE shows only a tiny periventricular lesion. However, an area of strong signal loss on T2-w was observed in the images of all EAE mice at 3 days after *H. polygyrus* infection, but this signal loss was weaker at six DPI (Fig. 2a). The resonance imaging was confirmed in a further post-mortem examination.

After imaging, the mice were assessed for lympho-mononuclear cell number in CSF and blood. Infection with larvae showed increased total cell number in the CSF and a reduced number of leukocytes in the blood. On Day 6, infection with L4 stage was associated with an impressive increase in total CSF cell number and cell viability in EAE mice (Fig. 2b, c). The Diff–Quik staining of CSF and blood leukocytes demonstrated significant increases in lymphocytes, monocytes and neutrophils in these mice at this time (Fig. 2d).

**Fig. 3:**
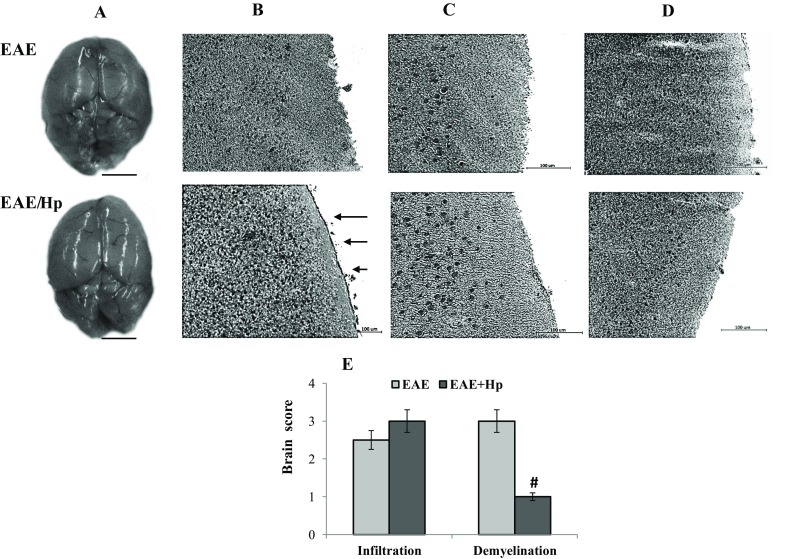
Morphology and histopathological changes of EAE mice at 6 days post-infection with *H. polygyrus*. Image of a representative morphology of mice brain given in coronal orientation (**a**, scale bar 5 mm). Frozen sections of brain were analyzed for hematoxylin–eosin staining (**b, c**) and solochrome cyanin (**d**) to detect leukocyte infiltration and demyelination, respectively. Leukocyte infiltration was enhanced in nematode-treated EAE mice (**b, c**). A large plaque of demyelination seen in the EAE mice was markedly attenuated by nematode treatment (**d**, magnified views of the boxed areas: scale bar 50 µm). Note the regeneration within the subdural space of infected mice (arrows). Differences in immunostaining intensities and colors result from tissue destruction. The brain pathology score was calculated by adding the infiltration or demyelination scores for each animal, and averaging the result within each group. The results of one representative experiment are presented as the mean ± SD of six mice. Statistical significance ^#^ *p* < 0.05 compared with EAE group was assessed by ANOVA

For histopathological analysis, the mice were assessed for brain cell infiltration on Day 6 post-infection. Brain lobe enhancement was evident in EAE-infected animals (Fig. 3a). As expected, microscopic alterations indicated that the number of leukocytes infiltrating the brain was enhanced in nematode-infected mice and the effect was paralleled by a decrease in the extent of demyelinated areas (Fig. 3b–e). Regeneration was observed within the subdural space of infected mice.

Brain fluid content was measured using the wet–dry method in separate experiments and was unchanged: 78 ± 4.6 and 80 ± 2% in uninfected and nematode-infected EAE mice, respectively.

### Nematode Effects on Blood–Brain Barrier Permeability

**Fig. 4:**
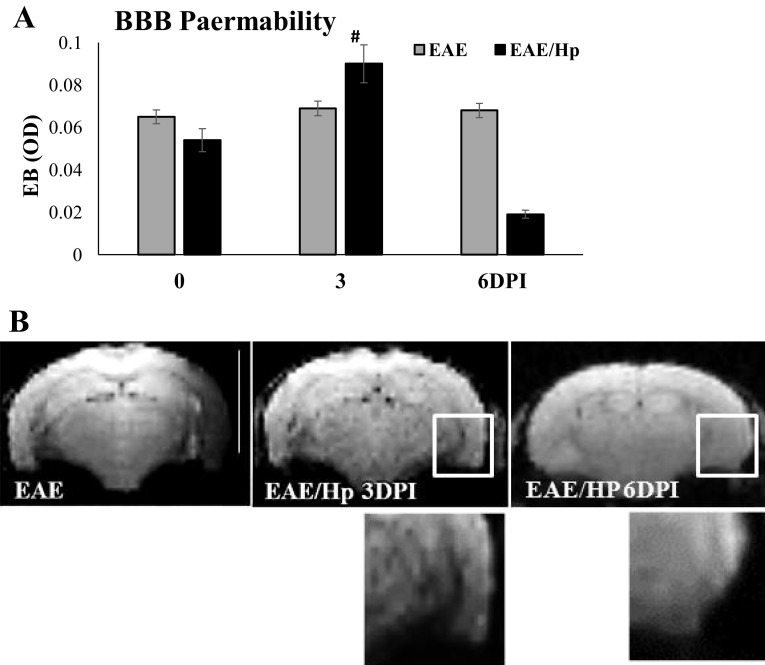
The effect of *H. polygyrus* infection on BBB permeability in EAE. BBB integrity was measured by Evans Blue extravasation (**a**). Mice were injected i.v. with 2% EB, were killed 30 min later, and the coloration of brain sections was assessed as an indicator of increased capillary permeability. The mean OD of extravased Evans Blue at day 0, 3, 6 is presented. To exclude the outside factor, the immunized group of mice was accompanied by a group of healthy animals and infected animals. Values are represented as means ± SD (*n* = 6 mice per group) from one representative experiment. Coronal T2-weighted images of the brains imaged 24 h after administration of superparamagnetic iron oxide particles—SPIO (**b**). Numerous signal-void regions seen on the macroscopic scale (in the box) as low signal intensities are related to the magnetic susceptibility effects caused by iron particles within phagocytic cells. The observation was carried on in a blinded fashion. Three animals per group were subjected to MRI at 3 and 6 days post-infection (6 DPI). Scale bar 5 mm

The integrity of the blood–brain barrier (BBB) was evaluated by quantitative measurement of EB: a common marker for albumin extravasation across the BBB (Wolman et al. 1981). The concentrations of extravasated EB in the different groups are shown in Fig. 4. *H. polygyrus*-treated EAE mice showed a significant increase in the content of EB in the brain at Day 3 post-infection. Subsequently, EB extravasation was reduced at Day 6 when the clinical signs of EAE were inhibited. In the control mice, baseline level of EB measured as OD was 0.3–0.5.

No significant differences were observed in BBB permeability after MOG antigen immunization in EAE mice compare to control, healthy mice, which is in line with the finding that migration of inflammatory cells to the nervous system is not necessarily associated with BBB opening (Rausch et al. 2003). No significant differences in brain weight (range 406–412 mg) were found between groups on each day of examination.

Information about the changes in the degree of BBB leakage was used to guide the choice of timing for the i.v. injection of iron oxide contrast agent in subsequent EAE animal imaging experiments. Cellular contrast FluidMAG-D–superparamagnetic iron oxide (SPIO) particles were used for the assessment of leukocyte (mainly macrophage) infiltration by magnetic resonance. In all EAE mice imaged on Day 3 post-infection, T2-weighted images obtained 24 h after SPIO injection displayed multiple focal areas of small abnormal regions of signal void corresponding to iron accumulation within multiple brain parenchyma regions (Fig. 4b). None of the control or infected mice showed abnormalities on Day 6.

### L4 Stage Effects on the CSF Inflammatory Milieu in EAE Mice

**Fig. 5:**
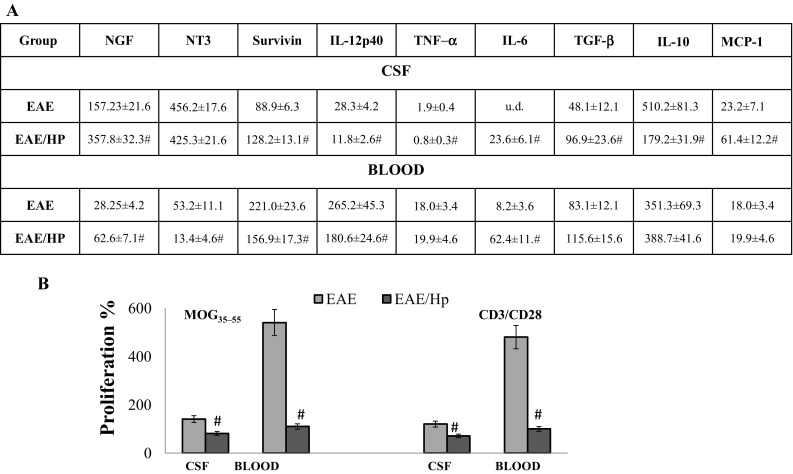
Activity of CSF and blood cells of EAE mice at 6 days post-nematode infection. NGF and neurotrophin NT3, survivin and cytokine levels in CSF and blood of the infected and uninfected EAE mice (**a**). Proliferative responses of cells upon stimulation with anti-CD3, costimulatory CD28 and with the encephalitogenic MOG_35–55_ peptide (**b**). Results represent the mean ± SD from six individual mice per group. Statistical significance ^#^ *p* < 0.05 between groups EAE and EAE/Hp treated in the same manner (in triplicate) was assessed by one-way ANOVA. *u.d*. under detection

To investigate changes in the levels of inflammatory factors, the CSF and blood of infected mice with EAE were assayed for IFN-γ, IL-4 IL-17A, IL-12p40, TNF-α, IL-6, MCP-1, IL-10, TGF-β, nerve growth factor (NGF) as well as NT3 and survivin concentrations on at Day 6 post-infection. Although the two groups of mice were broadly similar with regard to circulating and CSF IL-17A levels, an increase in TGF-β, MCP-1 and IL-6 was observed in the CSF of nematode-infected mice, and a decrease in IL-12p40, TNF-α and IL-10 levels (Fig. 5a). Very low levels of IFN-γ and IL-4 (range 1–10 pg/ml) were occasionally found in serum samples of nematode-infected and uninfected EAE mice (data not shown). The nematodes were found to interfere with NGF and survivin in EAE mice. Infection increased the NGF concentration in CSF and blood by approximately twofold but the NT3 neurotrophin level was unchanged in CSF and reduced in the blood. Interestingly, increased concentrations of survivin were found in the CSF of these mice (Fig. 5a).

### Antiproliferatory Effect of L4 Stage Infection

While high lymphocyte proliferation was observed in the blood and CSF in response to restimulation with immunizing MOG_35–55_ antigen and CD3/CD28 in EAE mice, the T cells of EAE mice treated with nematodes were not able to proliferate intensively (Fig. 5b).

### T Cells and Monocyte/Macrophage Phenotype of CNS Cells

#### T Cell Phenotype

**Fig. 6:**
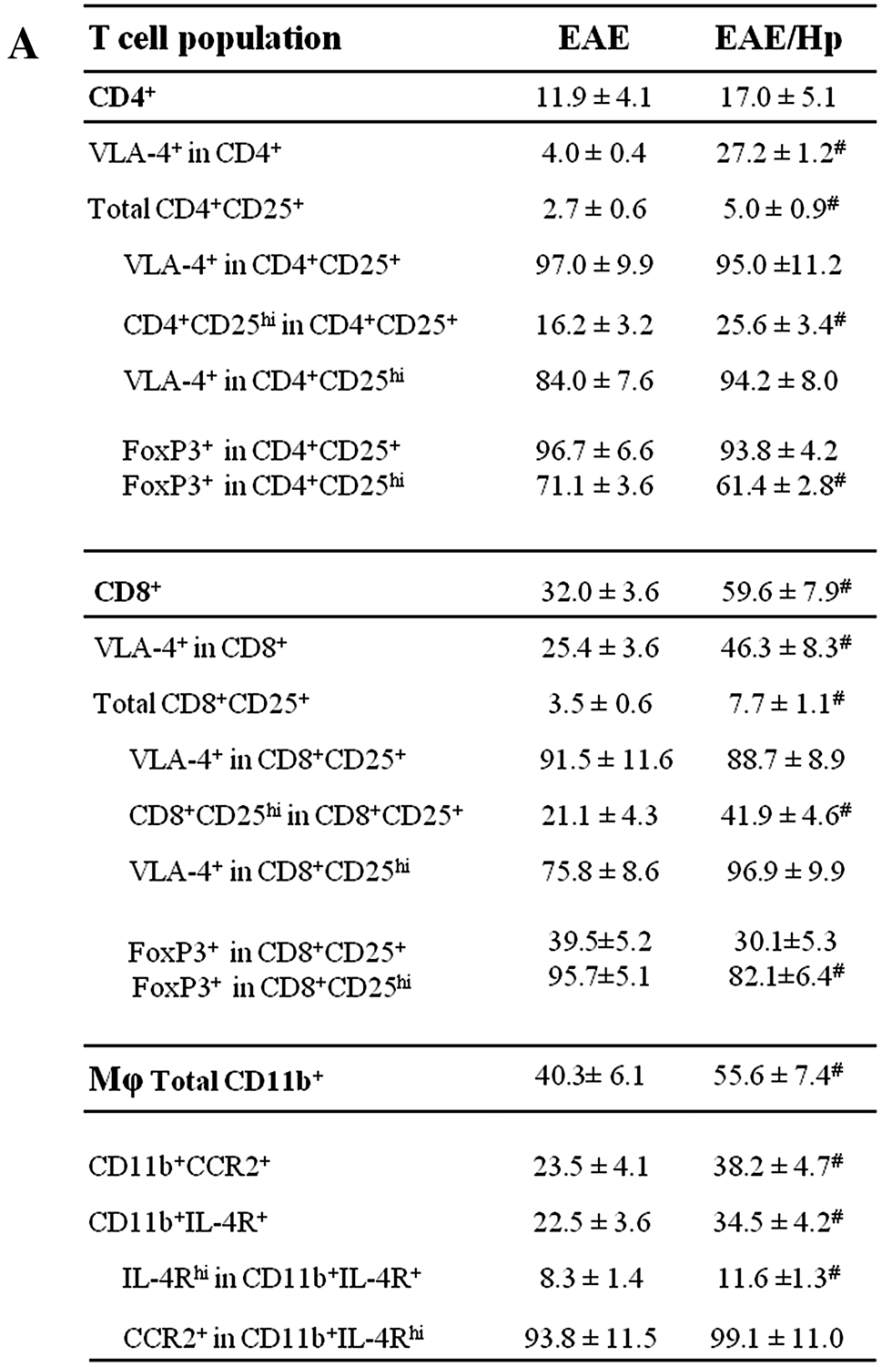

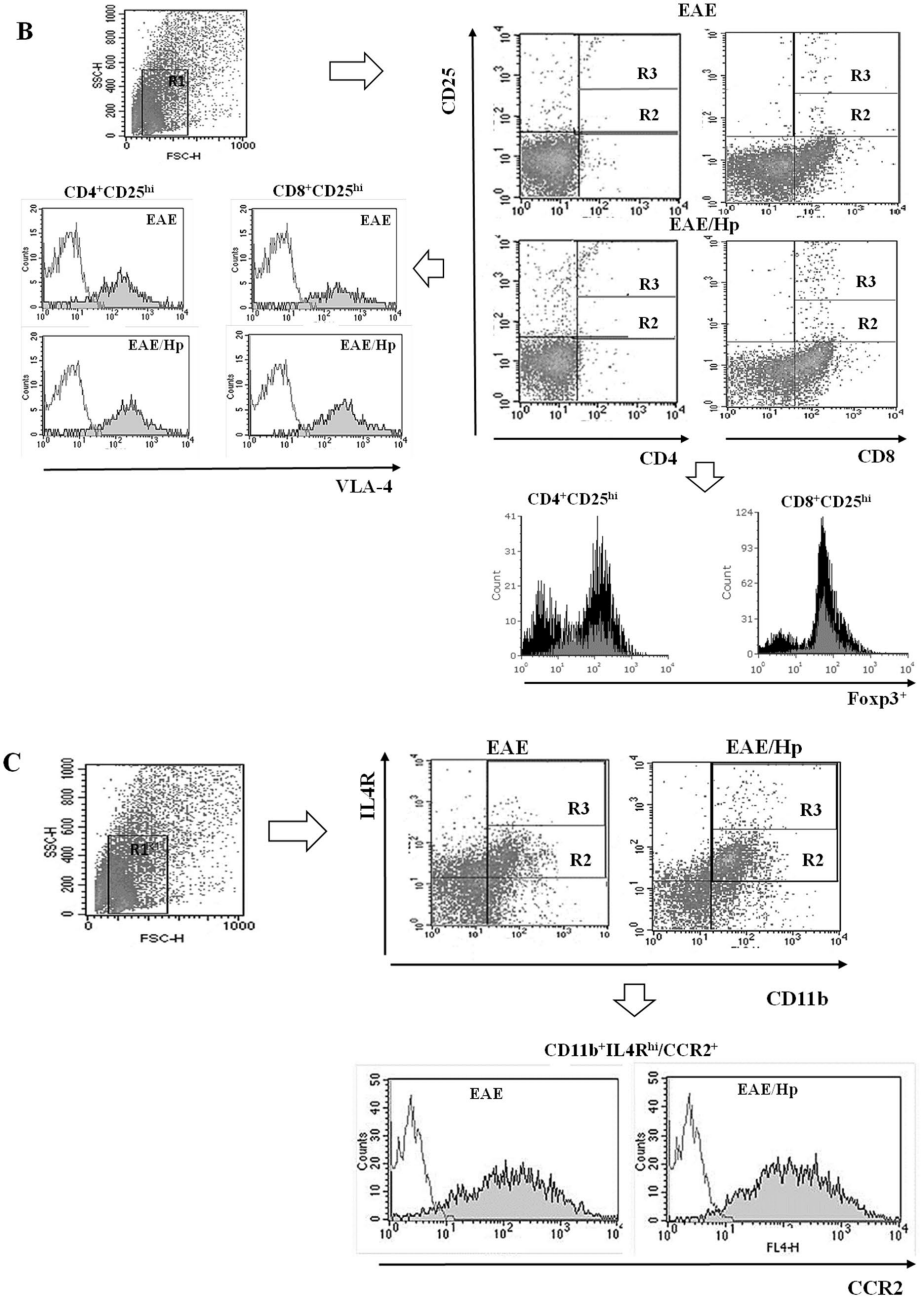
Expansion CD4^+^, CD8^+^ T cell subsets and CD11b-positive macrophages in CSF of EAE mice on Day 6 of *H. polygyrus* infection. T cell sub-population frequency was analyzed by flow cytometry. Results were presented as the frequency (%) of gated cells expressing the receptors CD4, CD8, CD25, Foxp3 and α4β1-integrin VLA-4 (**a**). Representative FACS plots analysis (**b**). Live leukocytes were initially gated based on their FSC/SSC distribution (R1) and further characterized for receptor expression. Right: representative FACS plot showing CD25^+^ (R2 + R3) or CD25^hi^ (R3) on CD4^+^/CD8^+^ (R3) profiles of gated cells. Left: histograms of gated R3 showing CD4^+^CD25^hi^ and CD8^+^CD25^hi^ cells with VLA-4 expression. Down: histograms of gated R3 showing CD4^+^CD25^hi^ and CD8^+^CD25^hi^ cells with Foxp3 expression for EAE (in gray) and for EAE/Hp (in black) mice. For CD11b-positive macrophages, the results are presented as frequency (%) of gated cells expressing CD11b, CD124 (IL-4Rα) and CCR2 (**c**). FACS plots showing representative frequency of CD11b^+^IL-4R^+^ (R2 + R3) and CD11b^+^IL-4R^hi^ (R3). Histograms of gated cells showing CD11b^+^IL-4R^hi^ cells with CCR2 expression. Flow analysis was performed on cells isolated from individual mice. The data are representative of one independent experiment. The results (**a**) represent the mean ± SD of six mice per group. Statistical significance ^#^ *p* < 0.05 was assessed by one-way ANOVA

Leukocytes were recovered from the CSF and peripheral blood and stained for surface markers as described in “Materials and methods” and the gating strategy presented in Fig. 6b, c. As expected, CD8^+^ T cells outnumbered CD4^+^ T cells in the inflammatory CNS and demonstrated expansion in the CSF of EAE mice (Fig. 6a). The CD4^+^ and CD8^+^ cell populations of EAE mice were affected by nematode infection. The total number of CD8^+^ T cells was greater than the total number of CD4^+^ T cells in the CSF, and greater frequencies were observed of CD4^+^ and CD8^+^ cells positive for CD25. These populations were found to have an increased percentage of CD4^+^CD25^hi^ and CD8^+^CD25^hi^ T cells, particularly the latter, demonstrating α4β1-integrin VLA-4 expression (Fig. 6a, b). Infection results in the percentage of CD4^+^CD25^+^Foxp3^+^ cells falling from 96.7% of all CD4^+^CD25^+^ cells in the CSF of EAE mice to 93.8%, and from 71.1% of all CD4^+^CD25^hi^ cells in the CSF of EAE mice to 61.4% (Fig. 6a, b). Due to the parallel expansion of CD25^+^Foxp3^−^ cells during infection, this results in a small reduction in the proportion of CD4^+^CD25^+^ cells which expresses Foxp3. The same effect was observed for CD8^+^CD25^+^Foxp3^+^cells. Infection results in the percentage of CD8^+^CD25^+^Foxp3^+^ cells falling from 39.5% of all CD8^+^CD25^+^ cells in the CSF of EAE mice to 30.1% and from 95.7% of all CD8^+^CD25^hi^ cells in the CSF of EAE mice to 82.5% (Fig. 6a, b).

In addition, no increase was observed in Foxp3 expression calculated based on median MFI within the Foxp3^+^CD4^+^CD25^+/hi^ and Foxp3^+^CD8^+^CD25^+/hi^ T cell population. No differences in the VLA-4 integrin expression on T cells were found.

#### Monocyte/Macrophage Phenotype

The CSF of infected EAE mice contained a higher proportion of macrophages with CD11b^+^ IL-4Rα^+^ including CD11b^+^ IL-4Rα^hi^; no significant changes were observed in CCR2 expression on these cells (Fig. 6a, c).

## Discussion

An in vivo MS prototype animal model of moderately severe ongoing monophasic EAE was established in female C57BL/6 mice. The model was induced by sensitization with myelin oligodendrocyte glycoprotein MOG_35–55_ peptide in CFA with *M. tuberculosis* H37RA and *B. pertussis* toxin PTX. During the effector phase of EAE disability, 3 weeks after sensitization, the mice were infected with 300 *H. polygyrus* L3. Like in previous studies (Donskow-Łysoniewska et al. 2012), the application of parasites resulted in almost complete recovery from neurological deficits at Day 6 post-infection, at a time when the L4 stage resides between the two muscle layers of the small intestine *muscularis externa*.

We found that the reduction in EAE symptoms observed in mice infected with the L4 stage was correlated with the presence of large numbers of leukocytes in the brain and in the CSF in contrast to the effect of adult *H. polygyrus* (data not shown).

CSF is in direct contact with the extracellular space and has been indicated in directing brain invasion by immune cells during EAE (Schmitt et al. 2012). These intriguing findings led us to closely examine lesion formation in EAE mice during the histotrophic phase of infection. Cellular infiltration is a hallmark of EAE models, and reductions in the severity of EAE are indicated by decreased clinical signs and CNS cellular infiltration. However, most studies, helminth administration took place prior to EAE induction as a form of pretreatment (La Flamme et al. 2003; Sewell et al. 2003).

In line with the previous observation that clinical disease signs correlate poorly with axonal loss in the CNS (Bjartmar et al. 2000), no clear lesions of EAE were observed in the brain under MRI examination in the present study. Areas of strong signal loss were observed in all MRI images from EAE mice on Day 6, and even more so on Day 3, post-*H. polygyrus* infection. On Day 3, BBB breakdown, as indicated by Evans Blue extravasation, was found to correlate with leukocyte infiltration, as confirmed by SPIO MRI. Although the mechanism remains unclear, this finding may indicate that the temporary BBB disturbance observed on Day 3 post-infection provides the leukocyte population unrestricted access to the CNS. As the L3 larvae present on Day 3 post-infection migrate by intestinal mucosa to the muscular layer, increases in BBB permeability and leukocyte migration can be provoked by tissue damage, inflammatory pain and gut microbiota changes occurring at this time (Braniste et al. 2014; Huber et al. 2001) or can be provoked by factors produced by immune cells: antigen-specific CD8 T cell proteins have been reported to disrupt the BBB (Lee et al. 2008; Suidan et al. 2008).

Interestingly, on Day 6 post-infection, when L4 larvae reside between the two muscle layers, the BBB was restored and a large number of leukocytes with a higher survival rate were localized in the CSF. At this time, the signs of EAE were found to be almost completely inhibited. Histological examination identified the infiltration of immune cells in the brain tissue of all mice treated with nematodes. This leukocyte infiltration was beneficial in reducing plaques. These changes were accompanied by reduced concentrations of IL-10, IL-12 and TNF-α, but increased concentrations of NGF (but not NT-3), as well as increased TGF-β, MCP-1 and survivin in the CSF. Although the localization of NGF in the brain must remain the subject of further research, our present findings indicated a twofold increase in expression of NGF in the CSF of mice infected with L4 stage nematodes. NGF neurotrophin can improve remyelination by promoting myelin repair in animal models of neuro-degenerative diseases (Althaus 2004), and has been shown to be critical for survival; the axonal growth of neurons also affects the regulation of proliferation, differentiation, neuro-transmission, and plasticity (Levi-Montalcini 2004). Interestingly, NGF is produced by CD4^+^ T cells, especially Th2 (Lambiase et al. 1997), and it interacts with the CD4^+^CD25^+^Foxp3^+^ T regulatory cells that suppress encephalitogenic T cells and inhibit EAE (Liu et al. 2006).

IL-10 is a promoter of strong Th2 responses in many helminth systems, and the expression of IL-10 is required to maintain EAE signs (Lee et al. 2008). Our results confirm those of previous studies (Wilson et al. 2005) which indicate that IL-10 does not appear to be a primary mechanism for helminth-associated Treg function, and in contrast to TGF-β, does not fulfill a purely down-regulatory role. These cytokine levels, in combination with a lack of Foxp3 induction on CD4^+^CD25^+^ T cells, indicated that *H. polygyrus* induced a regulatory immune response in the EAE mice, which has been previously reported for *H. polygyrus* single infection (Finney et al. 2007).

Treg are active at many points in the control of immune responses against parasites and are crucial for controlling the activity of self-reactive T cells: reductions in the numbers of these cells have previously been associated with their dysfunction in MS and EAE (Mills 2004). Importantly, Treg cells might migrate more rapidly across the brain endothelium than conventional T cells and accumulate in brain tissues (O’Connor et al. 2007).

Our findings indicate a very significant increase in the number of infiltrated CD4 and especially CD8 T cells with high expression of CD25 (IL-2Ra) in the nematode-treated EAE mice. This is the first study to confirm the participation of CD8^+^CD25^+^/^hi^ T cells in nematode intervention in EAE. In the CSF of EAE mice on Day 6 post-infection, 5% of CD4 and almost 8% of CD8^−^ cells were found express CD25, and approximately 25 and 42%, respectively, demonstrate high CD25 expression. This is a significant increase, given that physiologically 5–10% of CD4^−^ T cells and < 1% of CD8^−^ T cells from mice blood are CD25-positive, and the elimination of these cells produces autoimmune diseases (Sakaguchi et al. 1995). However, CD25 can also define non-regulatory T cells (Tconv) and effector T cells (Chaudhary et al. 2014), the phenotype shift observed in CSF lymphocyte induced by the nematodes in EAE mice resulted in the encephalitogenic T cells displaying a decreased proliferative response to MOG_35–55_ and TCR stimulation.

Further study is needed to determine the complete phenotype of these regulatory CD4 and CD8 T cells and the mechanisms behind the phenotype shift and immune suppression they induce, especially since additional cell types and other mechanisms besides TGF-β or CD4^+^CD25^+^FOXP3^+^ Treg cell induction are thought to provide crucial immunomodulation signals to the CNS (Fleming 2013).

The remission of EAE provoked by the nematodes was associated with enhanced proportion of macrophages, characterized by high expression of IL-4R; this is known to be associated with inhibition of the neuroinflammatory response due to these anti-inflammatory, suppressive, tissue-repairing and anti-proliferative abilities (Schwartz and Baruch 2014). Interestingly, very low amounts of IL-4 were detected in the mice studied herein, which is known to be required for alternatively activated macrophages (AAM*φ*) induction (Stein et al. 1992). These findings suggest that AAM*φ* CD11b^+^IL4R^hi^ can be activated locally by a nematode antigen and with definite phenotype can migrate to the CNS.

Nematode infection did not affect the expression of adhesion molecule VLA-4 on lymphocytes and of CCR2 on macrophages, and these receptors were highly expressed on leukocytes of all immunized mice. However, the persistence of the constant high receptor expression might allow and maintain transmigration of these immune cells. The change in CSF leukocyte phenotypes in the EAE mice infected with the nematode results in decreased proliferative response of encephalitogenic T cells to MOG_35–55_ and TCR stimulation.

In conclusion, our findings indicate that *H. polygyrus* L4 infection induces significant phenotypic changes in distinct subsets of CSF T cells and macrophages in EAE mice. Undoubtedly, functional studies are needed for further characterization of these regulatory cells and their mechanisms, but for now it is likely that many of the regulatory cells are adaptive cells with specificity for parasite antigens; they arise from precursors and migrate to the CNS when the blood brain barrier is temporarily disrupted, and resolve the inflammation associated with ongoing EAE. Together, these observations give an insight into the considerable role played by nematode infections in the regulation of the host environment and their effect on basic physiological processes.
